# *Salmonella* Typhi and *Plasmodium falciparum* Co-infection in a 12-year Old Girl with Haemoglobin E Trait from a Non-malarious Area in Bangladesh

**DOI:** 10.3329/jhpn.v28i5.6162

**Published:** 2010-10

**Authors:** Fahmida Chowdhury, Mohammod J. Chisti, Ahmadul H. Khan, Mohammad A. Chowdhury, Mark A.C. Pietroni

**Affiliations:** ^1^ Executive Director's Division; ^2^ Clinical Sciences Division, ICDDR,B, Mohakhali, Dhaka 1212, Bangladesh; ^3^ National Tuberculosis Control Programme, Mohakhali, Dhaka 1212, Bangladesh; ^4^ International Medical College, Gazipur, Bangladesh

**Keywords:** Haemoglobin E, Malaria, *Plasmodium falciparum*, *Salmonella* Typhi, Typhoid, Bangladesh

## Abstract

A 12-year old girl from Uttar Badda, Dhaka, Bangladesh, was admitted to the Dhaka Hospital of ICDDR,B, with a 23-day history of fever and diarrhoea. After admission, she was treated for culture-proven *Salmonella* Typhi-associated infection and was discovered to be heterozygous for haemoglobin E. Despite treatment with appropriate antibiotics, the patient's condition did not improve, prompting further investigation, which revealed malaria due to *Plasmodium falciparum*. Dhaka is considered a malaria-free zone, and the patient denied recent travel outside Dhaka. Subsequently, the patient recovered fully on antimalarial therapy.

## INTRODUCTION

Malaria is responsible for about one million deaths annually throughout the world (1). Although Africa accounts for 90% of the burden of mortality due to malaria, South-East Asia still suffers considerable mortality and morbidity. Of 11 countries of the World Health Organization in the South-East Asia region, 10 including Bangladesh are malaria-endemic (2). In Bangladesh, malaria is a major public-health problem in 13 of the 64 districts (3). Ninety-eight percent of malaria case reports come from 13 districts close to or bordering India and Myanmar (Fig.). Dhaka, the capital city of Bangladesh, is considered to be malaria-free. Three of the 13 districts—Bandarban, Khagrachari, and Rangamati collectively known as the Chittagong Hill Tracts (CHT) districts—report the highest incidence of malaria within the country (2–4). Approximately 50.6 million people (33.6% of the population) are at risk of malaria, of whom 10.9 million are at high or moderate risk, and 39.7 million are at low risk (3). In 2008, the numbers of confirmed malaria cases, probable malaria cases, and malaria-related mortality were 84,690, 83,972, and 154 respectively (3). The proportion of *Plasmodium falciparum* cases has been increasing at an alarming rate since 1999 and reached 83% in 2008 (3).

**Fig.**
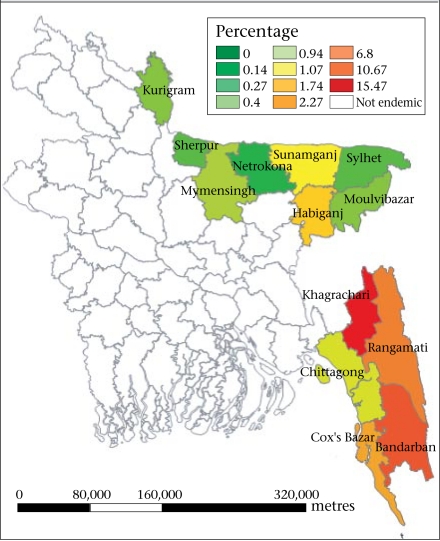
Map of Bangladesh showing malariaendemic areas

Malarial parasites reside in erythrocytes. Their optimal environment is in those that do not carry a haemoglobin variant. Haemoglobin E carriers are equally susceptible to malaria; however, the parasites are unable to multiply successfully in erythrocytes (5–7). This results in less severe disease and a lower risk of mortality. Haemoglobin E is one of the world's most common and important mutants and is common in South-East Asia where its prevalence can reach 30-40% in some parts of Thailand, Cambodia, and Laos. Haemoglobin E is also found in Sri Lanka, north-eastern India, Bangladesh, Pakistan, Nepal, Viet Nam, and Malaysia (6,7).

## CASE REPORT

A 12-year old girl was admitted to the Dhaka Hospital of ICDDR,B on 21 December 2009, with a history of fever for 23 days associated with chills and rigours which subsided with sweating after taking antipyretics, loss of appetite for 14 days, and passage of yellowish watery stool 7-8 times a day for five days. She lives with her family in Uttar Badda, Shaora bazaar, Dhaka. She and her mother reported that she had not travelled outside Dhaka for at least three months before the illness. She was treated with paracetamol and ciprofloxacin before attending the hospital. She had an uneventful birth-history and was fully immunized according to the national Expanded Programme on Immunization schedule.

On examination, she looked toxic and moderately pale; her temperature was 41 °C, pulse 90 per minute, blood pressure 100/70 mm of Hg, respiration rate 28 per minute, and tongue coated. On abdominal examination, her liver was palpable 2 cm below the right costal margin in the mid-clavicular line and was soft and non-tender. Her spleen was also palpable 2 cm below left costal margin along the long axis on deep inspiration and was soft in consistency. Other systemic examinations did not reveal any further abnormalities. A provisional diagnosis of acute watery diarrhoea with anaemia was made with a differential diagnosis of enteric fever.

Investigation revealed a haemoglobin of 9.5 g/L, total peripheral white blood cell count of 13,360/cmm, of which 67% were neutrophils, 27.5% lymphocytes, and 4.9% monocytes, and platelet count of 89,000/cmm. Blood culture grew *Salmonella* Typhi sensitive to ceftriaxone, rectal swab grew no organism, and Widal test report was TO (antibody against somatic antigen) 1:160, TH (antibody against flagellar antigen) 1:320. Peripheral blood film was reported as normocytic and normochromic; the Hb electrophoresis report revealed haemoglobin A to be 71.5%, haemoglobin E 25.3%, and haemoglobin A2 3%. Chest x-ray findings were normal, and ultra-sonogram of the whole abdomen showed mild hepato-splenomegaly. At that time, a diagnosis of typhoid fever with haemoglobin E trait was made. She was treated with ceftriaxone for 11 days, followed by azithromycin for seven days as she did not respond. Despite this, her temperature failed to settle. Antibiotics were stopped for 48 hours, and blood and urine cultures were sent again on the 21st hospital day. No growth of organisms was reported. ICT for kala-azar was negative. A blood-film showed trophozoites of *P. falciparum*. So, after three weeks of admission, a diagnosis of malaria due to *P. falciparum* was made. She was treated with oral quinine for seven days and became afebrile on the second day after starting treatment. She was discharged five days after starting quinine, completing the full course of treatment at home.

## DISCUSSION

*P. falciparum* is the most widespread and dangerous of the four *Plasmodium* species and can lead to fatal cerebral malaria if left untreated. In this case, it is interesting that our patient suffered from continuous fever due to *P. falciparum*-associated malaria for 44 days without treatment and did not yet deteriorate. It is evident from previous studies that *P. falciparum* grows slowly within both homozygous and heterozygous forms of haemoglobin E red blood cells (RBCs), which inhibits parasitaemia (5–9). The mechanisms underlying malaria protection by these genetic RBC variants have been studied extensively and can be classified into several broad groups: (a) reduced probability of merozoite invasion into the variant RBCs, (b) impairment of parasite growth within the variant RBCs, (c) enhanced removal of the parasitized variant RBCs, and (d) enhanced probability of infection early in life, particularly with *P. vivax*, which protects against subsequent severe malaria due to *P. falciparum* (10). We presume that our patient had a reduced severity of infection with *P. falciparum*-associated malaria as she was a carrier of haemoglobin E trait, although we cannot completely rule out the chance of this being a co-incidental finding.

Another interesting finding in this case is co-infection of *P. falciparum* with *Salmonella* Typhi. Because of the high prevalence of typhoid fever and malaria in the tropics, co-infections are not uncommon. However, the actual and precise underlying mechanism to explain the association between malaria and *Salmonella* spp.-associated infection is still uncertain, although it seems clear that malaria predisposes to bacteraemia and sepsis due to *Salmonella* (11). It has been shown that the antibody response to the O antigen of *S.* Typhi is markedly reduced in acute episodes of malaria compared to controls and that humoral immunity is transiently impaired (12). The signs and symptoms of malaria and typhoid fever often overlap. In a recent study, subjects with dual infection had significantly higher rates of nausea, vomiting, abdominal pain, and diarrhoea—the common features of enteric fever (13). In the last two decades, this relationship between the two diseases has been reported in studies from Africa and India (14–16).

Our patient was infected by malaria due to *P. falciparum* in Dhaka which is considered a malaria-free zone. Consequently, it is not usual for physicians in Dhaka to consider malaria in the differential diagnosis of patients with fever unless they have travelled to malarious areas. Thus, our case should alert health professionals to consider malaria in their differential diagnosis of similar cases. Nowadays, rapid diagnostic tests (RDTs) are available for malaria, with over 90% sensitivity and specificity whereas there is a chance of missing malarial parasites in microscopy, especially when the parasite count is low and when the microscopists are not used to seeing malaria. It would also be interesting to gather information from the main diagnostic centres and blood-banks in Dhaka that routinely perform RDTs for malaria to investigate the possibility that Dhaka is no longer malaria-free.
